# Benefits of Traditional Medicinal Plants to African Women’s Health: An Overview of the Literature

**DOI:** 10.3390/diseases13050160

**Published:** 2025-05-20

**Authors:** Fatiha Brahmi, Florence Kampemba Mujinga, Naima Guendouze, Khodir Madani, Lila Boulekbache, Pierre Duez

**Affiliations:** 1 Laboratory of Biomathemativs, Biochemistry, Biophysics and Scientometry, Faculty of Natural and Life Sciences, University of Bejaia, Bejaia 0600, Algeria; naima.guendouze@univ-bejaia.dz (N.G.); madani.khodir@univ-bejaia.dz (K.M.);; 2 Faculty of Agricultural Sciences, University of Lubumbashi, Lubumbashi B.P. 1825, Democratic Republic of the Congo; flokampemba@gmail.com; 3 Unit of Therapeutic Chemistry and Pharmacognosy, Université de Mons (UMONS), 7000 Mons, Belgium; 4 Agri-Food Technologies Research Center, Targua Ouzemmour Road, Bejaia 06000, Algeria; pierre.duez@umons.ac.be

**Keywords:** Africa, women’s diseases, medicinal plants, quality and safety, risks, guidance

## Abstract

**Background:** In many African areas, herbal products still represent a significant source of healthcare. However, a major gender bias is evident in the literature, as most of the work is carried out by male researchers, collecting data from male traditional practitioners, and thus often neglecting women’s specific health issues. This warrants a detailed review of the current knowledge about the major medicinal plants historically and still used for women’s health. **Objective:** This study aims to compile and critically analyze published data on the use of traditional herbal remedies by African women in addressing specific health conditions, in order to evaluate the potential of traditional medicine as a viable alternative or complementary approach to modern healthcare for women globally. **Methods:** Data were retrieved from databases by combining the following relevant keywords: “abortion, adverse, Africa, attendant, birth, botanical, delivery, developing, drug, ethnomedicine, ethnopharmacology, folk, gynecological, healing, infertility, herb, indigenous, lactation, medicine, native, obstetric, phytomedicine, plant, pregnancy, remedy, side, sub-Saharan, traditional, treatment, women”. **Results:** More than 125 studies, carried out across 12 African nations, revealed that up to 80% of African women resort to herbal medicines. An estimated 200 medicinally important plant species are reported to be utilized by women in different African countries, including Benin, Cameroon, Côte d’Ivoire, Egypt, Ethiopia, Ghana, Kenya, Mali, Nigeria, South Africa, Tanzania, and Zimbabwe. These herbs have many applications, mostly focused on infertility, pregnancy, painful menstruation, breast feeding, breast cancer, and contraception. Interestingly, according to their occurrence of usage, the plants most commonly reported for these conditions that are important to women are ambivalent plants (i.e., used both as foods and medicines) that include *Zingiber officinale* Roscoe, *Allium sativum* L., *Cucurbita pepo* L., and *Ricinus communis* L. **Conclusions:** Even though most women, in most African countries, do use traditional medicine, the amount of work published remains quite limited and no data are available in many countries. Therefore, it is desirable to expand African studies in this direction.

## 1. Introduction

In Africa, because health facilities are either too expensive, insufficiently developed, or remote, contemporary healthcare and medicine are frequently only accessible to a small number of people [1]; hence, herbal medicines significantly contribute to maintaining population health, both in rural and urban areas [2]. Continuously rising healthcare costs and an increase in both infectious and non-transmissible diseases make the situation even worse; different independent national surveys in the African region indicate that 90% of Ethiopians and Burundians, 85% of South Africans, 75% of Malians, and 70% of Rwandans, Beninese, and Ghanaians depend on medicinal plants [3]. The use of herbal remedies may also be influenced by societal and cultural factors, perceived efficacy and safety, and general accessibility [4] when the traditional healer-to-population ratio can be 100 times higher than the medical practitioner-to-population ratio [5]. In the majority of African countries, the inability to provide care that is accessible and of adequate quality is a major factor of unfavorable trends in women’s health indicators. This situation certainly results from a lack of investments made in women’s health [6], but other factors are also involved, such as the insufficient empowerment of women, inappropriate health systems, sociocultural behaviors, distance to medical facilities, and travel costs. Also, unsafe and/or illegal abortions still account for a large portion of women’s deaths [7].

Numerous studies indicate that both industrialized and developing nations have significant rates of concurrent herb-drug use; women and traditional practitioners around the world have employed many plants in similar indications that support women’s reproductive health. Of note, in many societies, gender roles will develop into particular kinds of knowledge, such as the responsibility of the family healthcare, and so women are the ones who developed practical knowledge about therapeutic plants [8]. Traditional remedies of most ethnic groups have helped to improve women’s healthcare, notably the treatment of menstruation troubles and gynecological diseases. For example, more than 570 such medications have been reportedly used in Asia, Europe, Oceania, Africa, and America to help women with their menstruation health [9].

In such a context, customary cultural practices play a most important role; traditional and folk medicines generally rely on plants that are most commonly collected in the wild and, to a smaller extent, cultivated. It should be noted that menstrual disorders, despite their impact on women’s daily activities, are typically not considered by global health organizations as a major health issue; an indignity, considering that the access to analgesics and/or sanitary facilities is often poor or nonexistent. Menstrual disorders are then often treated with many medicinal herbs of unproven efficacy and safety, which can result in physical symptoms linked to fertility loss [10].

African women also exploit plants to treat a variety of gynecological and obstetric issues, including infertility, pregnancy, lactation, cysts, and vaginal and womb cleansing. Despite their frequent use, the information on therapeutic plants for women’s reproductive health is generally scarce and has historically received little attention. Hence, only a few ethnobotanical surveys and limited literature reviews on *“women’s health issues”*, in relation with traditional herbal treatments, have been conducted in Africa. These investigations are generally dedicated to a few well-defined topics, notably pregnancy [4,11,12,13,14,15,16,17,18,19,20,21,22], infertility [7,23,24,25], breastfeeding [26], breast cancer [27], contraception [28], or maternity care [29,30,31].

So far, no review has been conducted to cover all specific feminine health problems treated by African medicinal plants and the studies carried out generally focus on specific countries. This highlights important research gaps, such as the dearth of data from numerous African nations and the requirement for more thorough studies on the safety and effectiveness of these conventional therapies, especially when used throughout vulnerable periods, such as pregnancy or menopause. And so, the present study aims to identify (i) which medicinal plants are used for diseases that compromise women’s health in African communities, and (ii) which safety measures could be proposed to ensure a successful and risk-free use of these plants. In a context of low-access to a modern health system, this research therefore examines the role of traditional medicines for women health, including desirable conditions for their rational and safe use.

## 2. Materials and Methods

**Figure 1:**
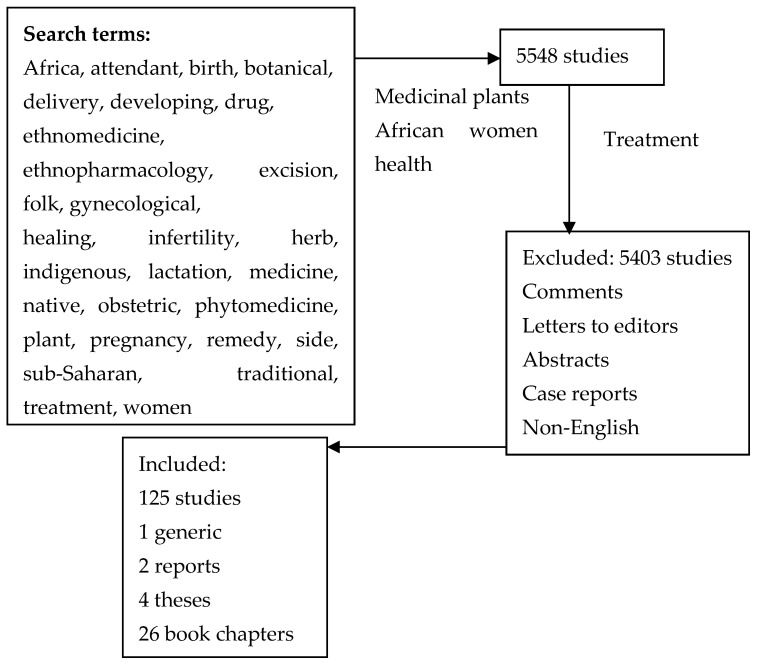
Search strategies for the current review.

From an extensive study of the literature, we attempted to collect data in different African countries on the use of plants by women as remedies for their health concerns. The tendencies of research were established from references recovered from PubMed, Scopus, Google Scholar, and Web of Science by combining the following research terms: abortion, adverse, Africa, attendant, birth, botanical, delivery, developing, drug, ethnomedicine, ethnopharmacology, excision, folk, gynecological, healing, infertility, herb, indigenous, lactation, medicine, native, obstetric, phytomedicine, plant, pregnancy, remedy, side, sub-Saharan, traditional, treatment, and women. The literature search covers the years 1946 to 2024. The reviewed papers were selected depending on how well they relate to the manuscript’s sections under consideration (Figure 1).

**Figure 2:**
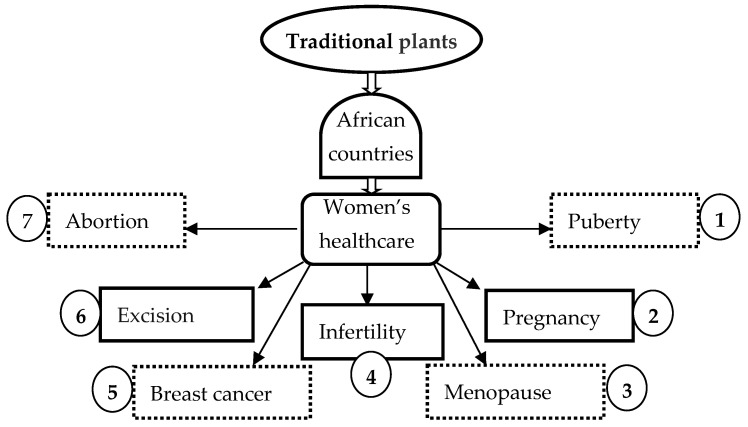
Concerns of African women treated using traditional plants such as those covered in the scientific literature.

The limited available data that we could retrieve confirm that the major health problems of African women have not been consistently addressed and so, only certain aspects could be approached. The present review then addresses the plants explored for menstrual disorders, maternity care, and reproductive healthcare problems, including infertility, contraception, pregnancy, and breast cancer (Figure 2).

## 3. Traditional Herbal Medicines for the Management of Major Diseases Affecting African Women

The diseases that are treated appear to be as varied as they are complex, and, in many cases, are gynecological in nature, i.e., disorders of the female reproductive system, including the ovaries, fallopian tubes, uterus, vagina and vulva, ovarian or vaginal cysts, uterine fibroids and prolapse. These can include reproductive tract infections (sexually transmitted, lower or upper genital) and anomalies, endometriosis, benign tumors or gynecological cancers (of the cervix, uterus, breast, ovaries, etc.). This Section discusses the most prevalent disorders about plants used by African women for their healthcare. The plants most reported are *Zingiber officinale* Roscoe (ginger), *Allium sativum* L. (garlic), *Cucurbita pepo* L. (pumpkin), and *Ricinus communis* L. (common castor bean).

### 3.1. Puberty

The onset of menstruation is followed by several symptoms; these can be painful, too abundant, or irregular. Distinctive authors report that, to relieve menstrual pain, African women use various natural remedies (Table 1).

In young girls, the color of white discharge can reveal intimate hygiene problems; in the case of brown or greenish discharge, some solutions have been passed down through the millennia, mostly from mother to daughter. Similarly, different countries have numerous methods of employing plants for this purpose (Table 1).

### 3.2. Pregnancy

**Figure 3:**
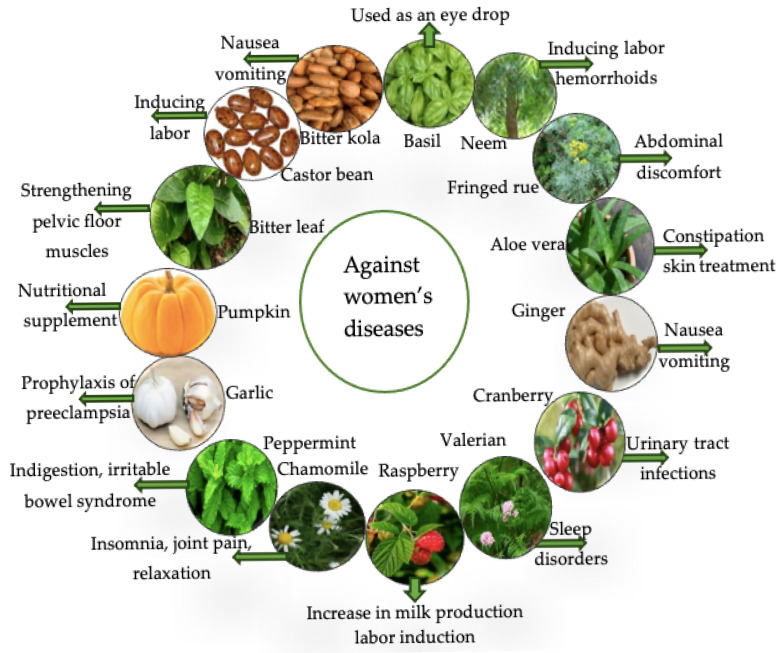
Main plants used by African women during pregnancy [18,47].

Over all of Africa, 4 out of 5 women are reported to turn to traditional medicine during pregnancy [13,45]. Herbal medications employed can be different, depending on the African locations and/or nations; the indications, that are part of maternal care, are either mother- or child-linked [46]. The common cold or flu, constipation, flatulence, gastrointestinal issues, pain conditions, improved fetal outcomes, prevention of miscarriage, reduction of anxiety, anemia treatment and/or prevention, and edema treatment are among the most frequently reported indications [18]. To discuss the involvement of plants at each stage of pregnancy, the most frequently used plants and their effects are summarized in Figure 3.

For morning sickness, in almost all African countries, women generally turn to ginger (*Zingiber officinale* Roscoe), a so-called *“miracle plant”* [45], taken as an infusion or chewed, to chamomile (*Chamomilla recutita* (L.) Rauschert, synonym of *Matricaria chamomilla* Blanco), and to fennel (*Foeniculum vulgare* Mill.).

Regarding edema, in Mali, women use an infusion of corn (*Zea mays* L.) to relieve edema during pregnancy [48].

Stomach burns are very frequent for some women; kongo bololo (*Chamomilla recutita* (L.) Rauschert, synonym of *Matricaria chamomilla* Blanco) is an important treatment in the D.R. Congo [49].

Concerning migraines, in the Maghreb, linden (*Tilia* spp.) is used to treat migraines [45]. To combat migraines and nervous tension in Egypt and Libya, women also use linden (*Tilia* sp.) [39,40,45,48].

To combat constipation in Egypt and Libya, women use linseed (*Linum usitatissimum* L.), which they leave to macerate in water overnight before drinking in the early hours of the morning [39,40,45,48]. Hemorrhoids are treated with a decoction or sitz bath of sulfur tree (*Morinda lucida* Benth.) in Burkina Faso, Ghana, Nigeria, Sierra Leone, and Togo [50].

As for the tocolytic effect, in the D.R. Congo, castor oil (*Ricinus communis* L.) is crushed and applied to the labia majora to close the cervix if it opens prematurely [51].

Infections are frequent during pregnancy since it is known as a period when a woman’s body is very vulnerable, and she is prone to a number of genital and urinary tract infections. In Ghana, Guinea-Conakry, Liberia, and Senegal, pregnant women apply to their genitals the crushed bark of the African lime tree (*Hallea stipulosa* (DC.) J.-F.Leroy, synonym of *Mitragyna stipulosa* Kuntze) [52,53].

Preparation for childbirth follows several rituals; during this period, many African women are surrounded by their loved ones and receive advice and care, generally from their grandmothers. Raspberry infusion (*Rubus idaeus* L.) is used for childbirth preparation in Senegal, Mali, Benin, Côte d’Ivoire, Niger, and Cameroon [40].

In preparation for breastfeeding, in Senegal, German chamomile (*Chamomilla recutita* (L.) Rauschert, synonym of *Matricaria chamomilla* Blanco) and calendula (*Calendula officinalis* L.) are ground and applied to the breasts [34].

For purgation, in the D.R. Congo, okra (*Abelmoschus esculentus* Moench) and fat grass (*Commelina diffusa* Burm.f.) are crushed and used to purge [54]. The same use is reported in Benin, Burkina Faso, and Côte d’Ivoire for kolatier (*Cola nitida* (Vent.) Schott & Endl.) and djeka (*Alchornea cordifolia* (Schumach.) Müll. Arg.) [55].

In Senegal, pelvic pain is alleviated through the use of tulip tree (*Spathodea campanulata* P. Beauv) [56].

### 3.3. During Labor and the Postpartum Recovery Period

After the arrival of the baby, there are a series of treatments that the woman must undergo.

#### 3.3.1. Expelling Placenta

In Ghana, Guinea-Bissau, Liberia, Nigeria, Senegal, and Sierra Leone, the infusion of wood stool (*Alstonia boonei* De Wild.) is often used to help expel the placenta [57]. A drink made from boiled leaves of Gambian tea (*Lippia multiflora* Moldenke) is also used [58,59].

#### 3.3.2. For Postpartum Hemorrhage

For postpartum hemorrhage, a treatment commonly reported in Burkina Faso, Guinea, Mali, and Nigeria consists of infused leaves of African red sandalwood (*Pterocarpus erinaceus* Poir.), kyama-guiguisuron (*Solanum torvum* Sw.) and stinkweed (*Cassia occidentalis* (L.) Rose and *Cassia caroliniana* Walter, both being synonyms of *Senna occidentalis* (L.) Link); this decoction is also used for fever resulting from pregnancy [36,39,40,45,48,60,61,62,63,64,65,66,67,68,69,70,71].

#### 3.3.3. For Postpartum Contractions

In the Eastern regions of Nigeria, the fruits of the Aridan tree (*Tetrapleura tetraptera* (Schumach. & Thonn.) Taub.) are used to prepare soups for nursing mothers from the first day of childbirth in order to prevent post-partum contractions [72].

#### 3.3.4. Help to Breastfeeding

Cameroon women who have just given birth drink and have their breasts massaged with a macerate of *Cassia polyacantha* Wild. Subsp. Campulacantha Hochst. Ex.A.Rich Brenan and *Phyllanthus muellerianus* (O. Kuntze) Exell. leaves [73]. According to Lockett et al. [74], the fruit extract of the desert date (*Balanites aegyptiaca* (L.) Delile) is added to porridge and eaten by nursing mothers to stimulate milk production in Burkina Faso, Ghana, Mali, and Senegal. In Egypt, Greek clove (*Trigonella foenum-graecum* L.) is boiled in water [75]. In Angola and Malawi, women are advised to drink carrot juice (*Daucus carota* L.) and basil (*Ocimum basilicum* L.) every morning to stimulate the milk production [40,48].

#### 3.3.5. Treatment of Genitalia

Throughout Africa, women are accustomed to applying *Aloe vera* (L.) Burm.f. gel after childbirth, or massaging the skin with olive oil (*Olea europaea* L.) once or twice a day [76]. In the D.R. Congo, castor oil (*Ricinus communis* L.) is introduced as a suppository into the vagina once a day to accelerate the shedding of the womb [77]. Plants can be involved at each stage of pregnancy; the most frequently used plants and the most significant ailments are summarized in Figure 3 and Table 2, respectively.

### 3.4. Menopause

In Morocco, Algeria, Egypt, and Libya, ginseng (*Panax ginseng* C.A. Mey.) [48], chaste tree (*Vitex agnus-castus* L.), and “clover” (*Chamaelirium luteum* (L.) A. Gray) are used to treat symptoms associated with this drop in estrogen and progesterone production. In the same region, women consider eating at least 10 g of oats for breakfast to help improve depression and low libido; also, sage infusion (*Salvia officinalis* L.) helps relieve hot flushes and night sweats [39].

### 3.5. Infertility

According to Telefo, Lienou, Yemele, Lemfack, Mouokeu, Goka, Tagne, and Moundipa [69], the problems of female infertility are solved in Ghana, Côte d’Ivoire, Nigeria, and Sierra Leone by the oral administration of 3 to 4 g of kwaonsuswaa fruit (*Solanum torvum* Sw.) macerated in palm wine. In Nigeria, Prekese (*Tetrapleura tetraptera* (Schumach. & Thonn.) Taub.) is reputed to be effective in the management of female sterility [72]. Pool [73] gives a list of plants that help in infertility treatment when administered as a decoction or infusion in the D.R. Congo. These include *Annona senegalensis* Pers., *Nelsonia canescens* Spreng, *Zanthoxylon chalybeum* Engl. *Amorphophalus abyssinicus* (A. Rich) N.E. Br., and *Phyllanthus muellerianus* (Kuntze) Exell.

### 3.6. Uterine Fibroids and Matrix Troubles

To treat uterine fibroids in Burkina Faso, Ghana, Mali, and Senegal, a zèkènè (*Balanites aegyptiaca* (L.) Delile) decoction is employed [78]; bubinga sap (*Guibourtia tessmannii* (Harms) J. Léonard) is used to purge every 2 days for a month; ginger (*Zingiber officinale* Roscoe) and garlic (*Allium sativum* L.) are crushed, added with a little water, mixed with aloe (*Aloes vera* (L.) Burm. f.) and honey and drunk [79]. In Libya, a mixture of houseleek (*Sempervivum* spp.) and honey is used [45,48]. Jaffré and de Sardan [51] list the various plants used in the D.R. Congo to “*solve matrix problems*”. Lengayamayi (*Ludwigia stenorraphe* (Brenan) H. Hara) is ground and applied to the genitalia to treat a poorly positioned or trackless matrix. Tona, Cimanga, Mesia, Musuamba, De Bruyne, Apers, Hernans, Van Miert, Pieters, and Totté [61] highlight some Congolese beliefs that “*making a decoction and drinking an infusion of bark from any tree whose roots cross a road will cure a curved womb*”.

### 3.7. Breast Cancer

There are not many African plant extracts specifically used for treating breast cancer, even though several of them have been found to exhibit anticancer action. For instance, *Tabernaemontana stapfiana Britten* (soccerball fruit) stem bark dried, ground into a powder, and combined with alcohol is used topically once per day for a month in Kenya. The dried leaves powder of *Glycine wightii* (Wight & Arn.) Verdc. (Synonym of *Neonotonia wightii* (Wight & Arn.) J.A. Lackey) (*perennial soybean*) is also applied topically. *Tragia brevipes* Pax (Climbing nettle) powder is infused and taken daily orally [80].

### 3.8. Abortion

In Cameroon, *Momordica charantia* L. (Bitter melon) is known for its abortive characteristic [81]. In West African markets, seeds from this plant are regularly offered for sale as abortifacients [82]; its fruit juice causes uterine hemorrhage, and the vine’s seeds have abortifacient properties [83]. Extracts of Tanzanian plants, including *Bidens pilosa* L. (Beggar’s Tick), *Commelina africana* L. (yellow commelina), *Desmodium barbatum* (L.) Benth (synonym of *Grona barbata* (L.) H. Ohashi & K. Ohashi) (hairy beggarweed), *Manihot esculenta Crantz*. (manioc), *Ocimum suave* Willd. (synonym of *Ocimum gratissimum subsp. gratissimum*), *Oldenlandia corymbosa* L. (two-flowered Oldenlandia), and *Sphaerogyne latifolia* Naudin (synonym of *Miconia platyphylla* (Benth.) L.O.Williams), have abortifacient effects since they induce strong uterine contractions [84]. In the north of Burkina Faso, drinking a watery solution made from the roots and leaves of *Securidaca longepedunculata* Fresen. (violet tree) supplies uterine contraction-stimulating ergoline alkaloids to induce abortions [85]. In Ghana, *Musanga cecropioides* R.Br. ex Tedlie (umbrella tree), *Erythrina senegalensis* DC. (Senegal coraltree), *Ficus sur* Forssk. (cape fig), and *Physalis angulata* L. (balloon cherry) are used as emmenagogues and to induce abortion [86]. A dose-dependent reversal of action may be implied by the fact that several species with a known spasmolytic activity (such as *Zingiber officinale* Roscoe and *Citrus* spp.) are also utilized to induce abortion, designed as “*uterine cleansing*” [10].

### 3.9. Excision

Some African nations still practice the age-old habit of female genital mutilation or female circumcision [87] but only scant information is available about plants used after the excision. To control any excessive bleeding, powdery mixtures of sugar, gum and herbs, ashes or pulverized animal manure are reported [88]. Birge and Serin [89] also indicate that a mixture of plants, cow dung, and butter have been used for wound healing while Nyangweso [90] stated that the circumciser pours some traditional herb on the wound, which causes “*excruciating pain*”. Only Vergiat [91] named the plants applied to the excision wound of women; these were recorded as the young shoots of *Ampelocissus cinnamochroa* Planch. (Synonym of *Ampelocissus bombycina* Planch.) and of *Hymenocardia acida* Tul.

**Table 2:** Plants used by African women for their healthcare.

Country	Plant Names	Usage/Indication	Reference
Benin	Violet tree (*Securidaca longipedunculata* Fresen.), *Buckbrush (Dichapetalum madagascariense* Poir.), *Tabaco cimarron* (*Schwenckia americana* L.), Sierra leone (*Pavetta corymbosa* F.N. Williams), African peach (*Sarcocephalus latifolius* (Sm.) E.A.Bruce), Spanish shawl (*Heterotis rotundi- folia* (Sm.) Jacq.-Fél.), Roundleaf sensitive pea (*Chamaecrista rotundifolia* (Pers.) Greene), Boundary tree (*Newbouldia laevis* (P. Beauv.) Seem)	Consumed during pregnancy: strengthening	[17]
Cameroon	*Lambe pundo* (*Senecio biafrae* (Oliv. & Hiern) C.Jeffrey.	For the treatment of female infertility, painful menstruations, horn’s gulps acid reflux	[69]
Cameroon	*a-guare-(a)nsra* (*Eremomastax speciosa* (Hochst.) Cufod.), Aframomum (*Aframomum letestuanum* Gagnep), White Weed (*Ageratum conyzoides* L.), Ooso (*Justicia insularis* T. Anderson), *West African aloe* (*Aloe buettneri* A. Berger)	For the treatment of female infertility	[69]
Cameroon	Lemongrass (*Cymbopogon citratus* (DC.) Stapf), Ragleaf, thickhead (*Crassocephalum bauchiense* (Hutch.) Milne-Redh.)	Swelling of legs and ankles, cleaning of the baby	[16]
Cameroon	*West African aloe (Morton)*	Swelling of legs and ankles, facilitate baby delivery, baby cleaning purposes, urogenital infections, postpartum abdominal pain.	[16]
Cameroon	Heartleaf fanpetals (*Sida veronicifolia* Lam.)	Swelling of legs and ankles or postpartum abdominal pain, in reducing pains of labor during or after childbirth, facilitation of delivery, body sweats, bleeding during pregnancy	[16]
Cameroon	Wandering jew (*Commelina benghalensis* L.)	Facilitate baby delivery, swelling of legs and ankles	[16]
Cameroon	*Roselle* (*Hibiscus noldeae* Baker f.)	Facilitate baby delivery, urogenital infections, postpartum abdominal pain; swelling of legs and ankles, cleaning of the baby	[16]
Cameroon	Neverdie (*Kalanchoe crenata* (Andrews) Haw.), Chinese hibiscus (*Hibiscus rosa- sinensis* L.)	Facilitate baby delivery	[16]
Côte d’Ivoire Nigeria	Scent leaf (*Ocimum gratissimum* L.)	Abdominal pain, loss of appetite, fever, cold and catarrh	[92,93]
Egypt	Peppermint (*Mentha × piperita* L.)	Gastrointestinal disorders, common cold, muscle pain, headache	[94,95]
Egypt	Anise (*Pimpinella anisum* L.)	Nausea, vomiting, abdominal colic	[94,95]
Egypt	Fenugreek (*Trigonella foenumgraecum*)	Stimulates uterine contractions, milk production, blood sugar levels reduction, stomachache	[94,95]
Egypt	Aniseed (*Anisum odoratum* Raf.), fenugreek (*Trigonella foenum-graecum* L.), Ginger (*Zingiber officinale* Roscoe), garlic (*Allium sativum* L.), green tea (*Camellia sinensis L. Kuntze*), and peppermint (*Mentha × pepirita* L.)	To treat abdominal colic during pregnancy, nausea, vomiting, and headache	[94]
Ethiopia	Ginger (*Zingiber officinale* Roscoe)	Pregnancy emesis, morning/motion sickness	[22,96]
Etiopia Nigeria	Garlic (*Allium sativum* L.)	Pregnancy symptoms	[22,97]
Ethiopia Nigeria	Pumpkins (*Cucurbita pepo* L.)	Nutritional supplement for pregnancy	[96,97]
Ethiopia	Fringed rue (*Ruta chalepensis* L.)	Nausea, vomiting, common cold, stomachache	[22,96]
Ethiopia	Eucalyptus (*Eucalyptus globulus* Labill.)	Fever, upset stomach, help loosen coughs	[22,98]
Ethiopia	Fringed rue (*Ruta chalepensis* L.), Eucalyptus (*Eucalyptus globulus* Labill.), Ginger (*Zingiber officinale* Roscoe), Garlic (*Allium sativum* L.)	Pregnant women: for treatment of nausea, morning sickness, vomiting, cough, nutritional deficiency	[98]
Ethiopia	Garlic (*Allium sativum* L.), Ginger (*Zingiber officinale* Roscoe), Fringed rue (*Ruta chalepensis* L.), Eucalyptus (*Eucalyptus globulus* Labill.), Basil (*Ocimum lamiifolium* Hochst)	Pregnant women: management of nausea, vomiting, abdominal pain, common cold, fever	[22]
Ethiopia	Ginger (*Zingiber officinale* Roscoe), Basil (*Ocimum lamiifolium* Hochst.)	Common cold, inflammation	[99]
Ethiopia	Ginger (*Zingiber officinale* Roscoe)	By pregnant mothers: morning sickness, cough during common old, aid digestion	[20]
Ethiopia	Garlic (*Allium Sativum* L.)	By pregnant mothers: common cold, flu, prevention of preeclampsia	[20]
Ethiopia	Basil (*Ocimum lamiifolium* Hochst.)	By pregnant mothers: headache, fever, hypertension, and flank pain	[20]
Ethiopia	Fringed rue (*Ruta chalepensis* L.)	By pregnant mothers: headache, fever, and cold	[20]
Ethiopia	Thyme (*Thymus schimperi* Ronniger)	By pregnant mothers: cough, stomach pain, and as a flavoring agent	[20]
Ghana	*Coffee senna* (*Senna occidentalis* (L.) Link), Common wireweed (*Sida acuta* Burm.f.) *Giant cola* (*Cola gigantea* A. Chev.)	To ease labor and improve fetal outcomes	[100]
Ghana	Groundnut tree (*Ricinodendron heudelotii* (Baill.) Pierre ex Heckel), Oil palm (*Elaeis guineensis* Jacq.), Spice tree (*Xylopia aethiopica* (Dunal) A. Rich.), Boundary tree (*Newbouldia laevis* (P. Beauv.) Seem, African tulip tree (*Spathodea campanulata* P. Beauv.), Giant sword fern (*Nephrolepis biserrata* (Sw.) Schott J.J.deWilde), African dragon tree (*Dracaena arborea* (Willd.) Link, Senegal Mahogany (*Khaya senegalensis* A. Juss)	Consumed during pregnancy: strengthening	[17]
Ghana	*Trade banayi* (*Trichilia monadelpha* (Thonn.), Bitter cassava (*Manihot esculenta* Crantz)	Consumed during pregnancy: antiemetic	[17]
Ghana	Black Afara (*Terminalia ivorensis* A.Chev)	Consumed during pregnancy: strengthening, to treat anemia	[17]
Kenya	Perennial soybean (*Glycine wightii* (Wight & Arn.) J.A. Lackey), Noseburn (*Tragia brevipes* Pax.)	To treat breast cancer	[80]
Kenya Nigeria	Castor oil (*Ricinus communis* L.)	Pregnant: delayed/protracted labor, retained after birth, postpartum hemorrhage, constipation and labor induction	[97,101]
Mali	*English tea bush* (*Lippia chevalierii* Moldenke.)	During pregnancy: well-being, nutrition or as a dietary supplement, common cold	[15,19]
Mali	Kinkeliba (*Combretum micranthum* G. Don)	During pregnancy: symptoms of malaria, edema, nausea	[15,19]
Mali	African locust bean (*Parkia biglobosa* (Jacq.) R.Br. ex G.Don)	During pregnancy: well-being, urinary tract infection, symptoms of malaria	[15,19]
Mali	*Guiziga*, *Mofou* (*Vepris heterophylla* (Engl.) Letouzey)	During pregnancy: symptoms of malaria, constipation, edema	[15,19]
Mali	*Nigerian stylo* (*Stylosanthes erecta* P.Beauv.)	During pregnancy: symptoms of malaria Tiredness	
Mali	Hog plum (*Ximenia americana* L.)	During pregnancy: well-being To increase appetite, heartburn	[15,19]
Mali	*English false abura* (*Mitragyna inermis* (Willd.) Kuntze)	During pregnancy: symptoms of malaria, Urinary tract infection	[15,19]
Mali	Dooki in Pulaar (*Combretum glutinosum* Perr. ex DC.)	During pregnancy: symptoms of malaria tiredness	[15,19]
Mali	*Manjanda marm* (*Opilia amentacea* Roxb.)	During pregnancy: Symptoms of malaria, to increase appetite, tiredness	[15,19]
Mali	*Kola tree* (*Cola cordifolia*)	During pregnancy: Well-being, symptoms of malaria, to increase appetite	[15]
Mali	Natal mahogany (*Trichilia emetica* Vahl)	During pregnancy: malaria tiredness	[15,19]
Morocco	Vervain (*Verbena officinalis* L.), cresson (*Nasturtium officinale R.Br.*), Madder (*Rubia tinctorum* L.), fenugreek (*Trigonella foenum-graecum* L.), Cinnamon (*Cinnamomum verum* J.Presl), ginger (*Zingiber officinale)*	Mothers who gave birth in the last 5 years: To get back in shape after delivery, facilitate child birth, vomiting, increase breast milk secretion	[102]
Nigeria	Bitter leaf (*Vernonia* *amygdalina* (Delile) Sch.Bip.)	Emesis, fever, constipation, loss of appetite during pregnancy	[92,103]
Nigeria	Bitter kola (*Garcinia kola* Heckel)	Nausea, vomiting	[97,104]
Nigeria	Nim tree (*Azadirachta indica* A. Juss.)	Pain, anemia, malaria, piles, make the unborn baby strong	[97,103]
Nigeria	Aloe vera (*Aloe vera* (L.) Burm.f.)	Pregnancy symptoms, skin treatment	[97,104]
Nigeria	Cannabis (*Cannabis sativa* L.)	Pain, fever, catarrh, loss of appetite, labor induction/speed up	[92,105]
Nigeria	Moringa (*Moringa oleifera* Lam.)	Fever, cough, emesis, helminths, diarrhea, diuretic	[97,104]
Nigeria	Camwood (*Baphia nitida* G. Lodd.)	Therapeutic meal	[93,97]
Nigeria	Palm kernel oil (*Elaeis guineensis* Jacq.)	Pregnancy symptoms	[103,104]
Nigeria	Gum arabic tree (*Acacia nilotica* (L.) Willd. ex)	Postpartum wound healing	[106]
Nigeria	Moshi medicine (*Guiera senegalensis* J.F.Gmel)	Nausea, vomiting, diarrhea and general well-being in maternal healthcare	[106]
Sierra Leone	*Angled luffa* (*Luffa acutangula* Roxb) ginger (*Zingiber officinale* Roscoe), lime (*Citrus aurantiifolia* (Christm.) Swingle)	Urinary tract infections, pedal edema, to improve fetal outcomes	[107]
South Africa	Dassiepis (*Hippobromus alata* Eckl. & Zeyh.)	Combined with Bakbos for cleansing after birth	[108]
South Africa	Moerbossie (*Anacampseros albissima Marloth)*	To cleanse womb after birth	[108]
South Africa	Isihlambezo	Quick and painless delivery, reduced vaginal discharge, reduce placental size, drain edema, fetal well-being	[109,110]
South Africa	Black monkey thorn (*Acacia burkei* Benth.)	To ease labor and labor pain	[7]
South Africa	Umpendulo (*Acalypha villicaulis* Hochst. ex A.Rich.)	During her first menstruation to shorten menstruation period, to regulate blood flow for next consecutive menses dysmenorrhea, and during pregnancy to ensure safe pregnancy and an uncomplicated delivery	[7]
South Africa	Creeping starbur (*Acanthospermum glabratum* (DC.) Wild)	To treat cervical pains during pregnancy	[7]
South Africa	Blue sweetberry (*Bridelia cathartica* Bertol.)	Dysmenorrhoea and infertility, to treat menorrhagia	[7]
South Africa	*Corkwood* (*Commiphora neglecta* I. Verd.)	To treat dysmenorrhea, menorrhagia, infertility, oligomenorrhoea, premature birth, and to cleanse the blood when pregnant	[7]
South Africa	Small-leaved Rattle-pod (*Crotalaria monteiroi* Trubert ex Baker f.)	To treat dysmenorrhea, menorrhagia, infertility, oligomenorrhoea, premature birth, and to cleanse the blood when pregnant	[7]
South Africa	Giant Dune Sedge (*Cyperus natalensis* Hochst. ex. C. Krauss)	To treat menorrhagia	[7]
South Africa	Hairy star-apple (*Doispyros villosa* (L.) De Winter)	For dysmenorrhoea	[7]
South Africa	*Dwarf coral tree* (*Erythrina humeana* Spreng.)	Dysmenorrhea and once a day for infertility; to prevent miscarriage as soon as a person knows they are pregnant	[7]
South Africa	*Natal guarri* (*Euclea natalensis* A.DC.)	Pregnant women for blood purification	[7]
South Africa	Common bushweed (*Flueggea virosa* Roxb. Ex Willd Royle)	For antepartum hemorrhage and to prevent premature birth.	[7]
South Africa	African Mangosteen (*Garcinia livingstonei* T. Anderson)	To treat dysmenorrhea and postpartum hemorrhage	[7]
South Africa	Crossberry (*Grewia occidentalis* L.)	To treat dysmenorrhoea, menorrhagia, infertility, oligomenorrhoea, premature birth, and to cleanse the blood when pregnant	[7]
South Africa	Red spikethorn (*Gymnosporia senegalensis* Loes.)	Used as an enema and to treat infertility	[7]
South Africa	*Gombossie* (*Hermannia boraginiflora* Hook.)	To treat dysmenorrhoea and during pregnancy to ease labor	[7]
South Africa	*Lala palm* (*Hyphaene coriacea* Gaertn.)	For dysmenorrhoea, infertility, after birth pains, postpartum bleeding, and to ease labor	[7]
South Africa	*Qtar-grass* (*Hypoxis* cf. *longifolia* Baker)	To treat menorrhagia	[7]
South Africa	*Star flower* (*Hypoxis hemerocallidea* Fisch., C.A.Mey. & Avé-Lall.)	For dysmenorrhagia	[7]
South Africa	Sausage tree (*Kigelia africana* (Lam.) Benth.)	For blood cleansing and pelvic pains during pregnancy	[7]
South Africa	Showy plane (*Ochna natalitia* Walp.)	To treat dysmenorrhoea, menorrhagia and infertility taken for after-birth pain. To ease labor and reduce delivery complications. To treat menorrhagia, dysmenorrhoea and for blood purification in a pregnant woman.	[7]
South Africa	Erect prickly pea (*Opuntia stricta* (Haw.) Haw.	Pregnant at the third trimester to dilate the cervix and to cleanse blood	[7]
South Africa	Weeping resintree (*Ozoroa engleri* R.Fern & A. Fern.	To treat dysmenorrhoea and after birth pains	[7]
South Africa	Rhodesian blackwood (*Peltophorum africanum* Sond.)	For dysmenorrhoea and blood cleansing when pregnant.	[7]
South Africa	Wild Buttercup (*Ranunculus multifidus* Forssk.)	To treat infertility, to cleanse blood when pregnant and to ease labor	[7]
South Africa	Baboon grape (*Rhoicissus digitata* Gilg & M.Brandt)	To treat dysmenorrhoea and infertility, amenorrhoea.	[7]
South Africa	*Sapium integerrimum* Hochst.	For menstruation and dysmenorrhoea	[7]
South Africa	Jelly plum (*Sclerocarya birrea* Hochst.)	For pregnancy to induce abortion	[7]
South Africa	Hairy coastal currant (*Searsia nebulosa* (Schönland) Moffett)	For dysmenorrhoea and infertility	[7]
South Africa	Canary creeper (*Senecio deltoideus* Less)	To treat infertility.	[7]
South Africa	Ichazampuk-ane (*Senecio serratuloides* DC.)	To treat infertility, to cleanse blood when pregnant and to ease labor	[7]
South Africa	Toad tree (*Tabernaemontana elegans* Stapf)	Dysmenorrhoea and infertility	[7]
South Africa	Natal mahogany (*Trichilia emetica* Vahl)	To induce abortion at a first trimester of pregnancy used to massage the belly of a woman during labor and reduce pains	[7]
South Africa	Heart-leaved brooms and brushes (*Acalypha brachiata* Krauss)	For contraception	[111]
South Africa	Bitter aloe, tap aloe, red aloe (*Aloe* spp.)	For contraception	[112]
South Africa	Holy thistle (*Centaurea benedicta* (L.) L.)	For contraception	[113]
South Africa	Blue bitter-tea (*Gymnanthemum myrianthum* (Hook.f.) H. Rob)	For contraception	[114]
South Africa	Bitter karkoe (*Kedrostis nana* Cogn.)	For contraception	[115]
South Africa	Hell-fire bean (*Mucuna coriacea* Baker)	For contraception	[116]
South Africa	Sausage tree (*Kigelia africana* (Lam.) Benth.)	For contraception	[7]
South Africa	Soap-nettle (*Pouzolzia mixta* Solms)	For contraception	[117]
South Africa	Cauliflower (*Salsola tuberculatiformis* Botsch.)	For contraception	[118]
South Africa	Dwarf Mexican (*Schkuhria pinnata* (Lam.) Kuntze	For contraception	[117]
South Africa	Violet tree (*Securidaca longepedunculata* Fresen.)	For contraception	[114]
South Africa	Marula (*Sclerocarya Birrea* Hochst.)	Female infertility	[23]
South Africa	Beggar’s tick (*Bidens pilosa* L.)	Menstrual disorder	[23]
South Africa	Coast silver oak (*Brachylaena discolor* DC.)	Female infertility	[23]
South Africa	Misbeksiektebos (*Geigeria aspera* Harv.)	Period pains	[23]
South Africa	Paintbrush flower (*Kleinia longiflora* DC.)	Menstrual disorder	[23]
South Africa	Pawpaw (*Carica papaya* L.)	Abortion	[23]
South Africa	Bushveld saffron (*Elaeodendron* *transvaalense* (Burtt Davy) R.H.Archer)	Female infertility	[23]
South Africa	Paper reed (*Cyperus papyrus* L.)	Menstrual disorder	[23]
South Africa	Prostrate Sandmat (*Chamaesyce* *Prostrata* Aiton)	Womb problem	[23]
South Africa	Candelabra tree (*Euphorbia ingens* E.Mey. ex Boiss.)	Breast cancer	[23]
South Africa	Purging nut (*Jatropha curcas* L.)	Impotence, vaginal candidiasis	[23]
South Africa	Cork bush (*Mundulea Sericea* (Wild.) A.Chev.)	Menstrual disorders	[23]
South Africa	African blackwood (*Peltophorum africanum* Sond.)	Female infertility, post-partum	[23]
South Africa	Spanish gold (*Sesbania punicea* (Cav.) Benth.), Lavender tree (*Heteropyxis Transvaalensis* Schinz.)	Menstrual disorder	[23]
South Africa	*Satin squill* (*Drimia elata* Jacq. ex Willd.)	Female infertility; impotence	[23]
South Africa	*Star-grass* (*Hypoxis obtusa* Burch.)	Female infertility	[23]
South Africa	African soapberry (*Phytolacca dodecandra* L’Hér.)	Female infertility; menstrual disorder	[23]
South Africa	African sandalwood (*Osyris lanceolata* Hochst. & Steud.)	Impotence; menstrual disorder	[23]
South Africa	Habanero pepper (*Capsicum chinense* Jacq.)	Period pains	[23]
South Africa	Hell-fire bean (*Mucuna coriacea* Baker.	For contraception	[116]
South Africa	Sausage tree (*Kigelia africana* (Lam.) Benth.)	For contraception	[7]
South Africa	Soap-nettle (*Pouzolzia mixta* Solms), Dwarf Mexican (*Schkuhria pinnata* (Lam.) Kuntze ex Thell.)	For contraception	[117]
	Cauliflower (*Salsola tuberculatiformis* Botsch.), Saltwort	For contraception	[118]
South Africa	Violet tree (*Securidaca longipedunculata* Fresen.)	For contraception	[114]
Zimbabwe	*Snuggle-leaf* (*Pouzolzia mixta* Sohms) Okra (*Abelmoschus esculentus* Moench)	For widening of birth canal, labor induction, nutritional supplement	[105]
Zimbabwe	Makoni (*Fadogia ancylantha* Schweinf.) Tea Bush (*Abelmoschus esculentus* Moench) Okra Chir pine (*Pinus roxburghii* Sarg.)	To facilitate childbirth, for *“widening of birth canal”*	[119]

## 4. Scientific Evidence for the Efficacy of the Most Reported Herbal Remedies (Castor Bean, Garlic, Ginger, Pumpkin)

There is limited scientific evidence about the effects of most medicinal herbs on the female body and more research is definitely needed to elucidate the active compounds and mechanisms of action for reported uses.

### 4.1. Castor Bean

A proprietary *Ricinus communis* L. non-polar seed extract is reported to present anti-implantation, contraceptive, and estrogenic activity in rats and mice, acting at multiple sites, including the oviduct, fallopian tube, uterus, and the endometrial implantation site, and disrupting the estrogen/progesterone cycle [120]. The seed and petroleum ether fraction (5–20 mg/kg) showed high antifertility efficacy in both animals and in women volunteers [121]. Another *R. communis* seed extract inhibited both steroid releases and suppressed the stimulatory effect of LH on progesterone release [122]. The seed oil may prevent women from ovulating in part by impeding the growth of follicles or through an estrogen-induced hyperprolactinaemic hypogonadal mechanism [123]. This effect may also be due to alterations in uterine smooth muscle quiescence and inertia [124]. Ricinoleic acid and sterols may be the cause of the altered lipid profiles, hormonal balance, and uterine histological changes observed in pregnant rats during the early stages of gestation [125]. *R. communis* fruit extract inhibits migration/invasion, induces apoptosis in breast cancer cells, and arrests tumor progression in vivo. These effects could be related to the alkaloid ricinine, to *p*-coumaric acid, epigallocatechin and/or ricinoleic acid [126].

### 4.2. Garlic

*Allium sativum* L. is a natural remedy for various women’s ailments, reported to help regulate hormones, improve fertility, decrease menstrual pain, control the menstrual cycle, and diminish inflammation in the female reproductive system. Garlic contains a volatile oil (0.1–0.36%) with a complex mixture of sulfur compounds, notably diallyl sulfide and alliin, decomposed into allicin by the enzyme alliinase. Allicin is reported to modulate hormone levels and to present antioxidant and anti-inflammatory properties; other antioxidants of garlic include vitamin C and selenium that could protect the ovum from oxidative damage. Diallyl disulfide was found to stimulate the anterior pituitary gland, increasing the secretion of the luteinizing hormone, and raising the basophil count [127]. Garlic may be beneficial for post-menopausal women as it yields a partial recovery in serum estrogen titer; a garlic oil supplementation in bilaterally ovariectomized rats is consistently linked to improved bone mineral content preservation and increased calcium transference [128].

### 4.3. Ginger

*Zingiber officinale* Roscoe has been studied mainly in pregnancy for reducing nausea and vomiting, either fresh or as a powder, essence, or extract, with doses ranging from 0.5 to 2.5 g/day [129]. Ginger is also reported as a natural remedy for dysmenorrhea and to manage the intensity of labor pain due to its content of gingerols, analgesic and anti-inflammatory cyclooxygenase (COX-2) inhibitors [130] and blockers of the coding genes that promote the synthesis and secretion of pro-inflammatory cytokines in the inflammatory region [131].

### 4.4. Pumpkin

*Cucurbita pepo* L. seed may have estrogenic modulatory properties [132], possibly due to its high content in phytoestrogen substances with estrogenic-like properties, such as lariciresinol and secoisolariciresinol [133]. Pumpkin seed extract was reported to mitigate menopause-related disorders in ovariectomized rats through the improvement of lipid profiles, the reduction of oxidative stress and thermogenesis, and the increase in alkaline phosphatase activity [134].

## 5. Risks Possibly Associated with Herbal Treatments for African Women

### 5.1. Quality and Safety Concerns

Many rural dwellers in Africa believe that, since their ancestors “*used herbal blends/concoctions for their well-being in the past and with no adverse effects*”, they can assume that, as herbal blends are “*natural*”, their safety is necessarily guaranteed [135]. However, in most African countries, traditional healers and midwifes lack regulatory oversight to guide their practices and products. As the use and marketing of herbals are most often practiced without evaluation of quality and possible risks [136], a number of adverse effects are obviously associated with the use of some low-quality, adulterated, contaminated, mislabeled, or toxic products that have posed and still pose, in some African regions, serious threats to public health [137,138].

### 5.2. Self-Medication and Lack of Information on the Use of Plants

Although products can be purchased from an herbalist or prescribed by a traditional practitioner or midwife, household access to medicinal plants may consist of the direct collection of products *in natura*, a practice based on traditional popular knowledge, acquired from generation to generation and generally referred to as “*folk medicine*”. Self-medication is then a common habit among people living in rural and semi-urban communities in African countries as they usually have easy access to plant material [137,139]. However, at the exception of a few very popular neighboring plants, local knowledge is generally limited; confusions in botanical identity, collected organ, collection conditions (season, location, etc.), modes of preparation, disorder diagnosis, drug dosage, and posology are possible [138], increasing the risk of adverse effects, especially in vulnerable groups such as the elderly, children, and pregnant women. Also, mixtures of plants are often prepared without exact knowledge of each plant’s pharmaco-toxicology or of the possible interactions that may arise from its combination [137]. For example, the use of soy-based products raises questions about hormonal effects of their phytoestrogens and possible interference with gestational cycles. Isoflavones from soy induce mammary gland hyperplasia and renal tubule calcification in female animals [140].

### 5.3. Risks of Over- or Under-Dosage and Toxicity

Traditional healers are often very secretive about their practices and their prescriptions can be quite vague, sometimes resulting in over- or under-dosages, especially as no regulatory body controls the standardization or use of herbals [18]. Over- and under-dosages may lead to short-, mid-, and long-term adverse/toxic effects (including cardio-, neuro-, hepato-, nephro-, genotoxicities) and treatment failures [141]; these risks are amplified by patient-related factors, such as co-morbidities, and renal or hepatic impairments [138].

### 5.4. Risks Associated with the Use of Plants by Pregnant Women

As stated in Section 3.2, African women tend to turn to natural herbal medicines rather than prescription drugs to deal with pregnancy troubles, but also to “*ensure a healthy development of the fetus*” [15,18,47,142]. The risks and long-term negative health effects involve both maternal and neonatal morbidity and mortality. For example, side effects have been associated with the use of fenugreek (*Trigonella foenum-graecum* L.), harmel (*Peganum harmala* L.), nigella (*Nigella sativa* L.), rosemary (*Rosmarinus officinalis* L.), or *Artemisia herba-alba* Asso. (Synonym of *Seriphidium herba-alba* (Asso) Y.R. Ling), which are all popular pregnancy herbals in Morocco [142]. For some of these plants, these adverse effects are well-documented. Regarding fenugreek, despite that fact that it could enhance milk ejection by stimulating the secretion of oxytocin and prolong the duration of peak milk synthesis by modifying the insulin/GH/IGF-1 axis [143], the administration of its seeds significantly increase pituitary oxytocin expression and plasma insulin concentration, with a risk of uterine contractions and hypoglycaemia [94]. *Peganum harmala’s* different organs contain 1.7–5% of b-carboline alkaloids [144], known serotonin agonists and central anticholinergic agents (Achour et al., 2012); the poisoning of two pregnant women in Morocco led to uterine hypertonicity at term in one patient and to placental abruption in the other [145]. In animal studies, *Nigella sativa* seed extracts induce a significant decrease in fetal survival rates [146] while their major compound, thymoquinone, affects embryonic development [147]. Rosemary extracts are suspected of abortive effects [148]; in animal studies, an aqueous extract was associated with a possible anti-implantation effect without interfering with the normal developments of the concept after implantation [149]. In animal studies, transplacental exposure to *A. herba-alba* was found to increase infertility, delay memory function, and neuromotor reflex in offspring mice; this was ascribed to the plant content in isoflavones [150] that exhibit both estrogen-mimetic and anti-estrogen activity that may contribute to infertility and reproductive abnormalities [151]. In a major review study, Bernstein et al. [152] reported a list of other plants to avoid during pregnancy: *Abrus precatorius* L., *Achyranthes aspera* L., *Ailanthus excelsus* Roxb., *Aloe vera* (L.) Burm.f., *Aristolochia indica* L., *Areca catechu* L., *Bambusa vulgaris* Schrad. ex J.C. Wendl., *Cassia occidentalis* L., *Cicer arietinum* L., *Cimicifuga racemose* (L.) Nutt. (*Synonym of Actaea racemosa* L.), *Dolichandrone falcata* (Wall. ex DC.) Seem., *Ginkgo biloba* L., *Hydrastis canadensis* L., *Indigofera trifoliata* L., *Lavandula latifolia* Medik., *Maytenus ilicifolia* Mart. ex Reissek, *Momordica cymbalaria* Fenzl ex Naudin, *Moringa oleifera* Lam., *Musa rosacea* Jacq. (Synonym of *Musa balbisiana var. balbisiana*), *Oxalis corniculata* L., *Phytolacca dodecandra* L’Hér., *Plumeria rubra* L., *Ricinus communis* L., *Ruta graveolens* L., *Stachys lavandulifolia* Vahl, *Senna alata* (L.) Roxb., *Trigonella foenum-graecum* L., *Vitus agnus-castus* L., and *Valeriana officinalis* L. This list gives precious indications for evaluating safety measures to be considered with related species. Table 3 repertories the known side effects of some of the medicinal plants used in Africa by pregnant women.

### 5.5. Suggestions for Guidance

There appears to be a serious need to develop the awareness of the potential health risks associated with the use or abuse of herbals, especially for women who are particularly at risk in crucial periods, including during menstruation, pregnancy, and menopause.

#### 5.5.1. Quality Aspects

Because of issues with identity, purity, strength, and performance qualities, quality control is one of the most important factors in determining the safety and toxicity of a certain plant. Incorrect plant components, contaminants such pesticides and pollutants, hazardous metals, bacteria, molds, and mycotoxins, and processing impurities are examples of purity problems [168]. Masullo et al. [169] emphasize the necessity of critically assessing the effectiveness, safety, and quality of medicinal plants used for women’s health using analytical methods. For the identification of active/toxic compounds and the quality control of herbal items that contain a lot of different, low-concentration unknown chemicals, fingerprint analysis could be a straightforward method.

#### 5.5.2. Particular Precautions for Pregnancy and Menopause

According to Stephens [170], pregnant women should (i) be made aware of the potential risks of contaminants; (ii) be urged not to take unregulated drugs before and during pregnancy; (iii) obtain informed (medical/pharmaceutical) advice before using any herbal, considering the dosage, duration of use, mode of administration, and timing, with the first trimester being particularly at risk for teratogenesis. Many herbs used to treat menopausal symptoms might have unfavorable side effects such as gastrointestinal disorders (*Medicago sativa* L., *Hypericum perforatum* L., *Glycine soja* Sieb.) [171] and skin and subcutaneous tissue diseases (*Glycine max* (L.)) [168]. When *Salvia officinalis* L. is used excessively for managing the symptoms of menopause, it might result in tachycardia, fever, disorientation, and convulsions that resemble epilepsy [171]. As general precautions, concentrated extracts, high amounts of herbal compounds, and/or extended use should be avoided [170]. With such safety precautions being obviously difficult to apply in a rural African context, a training of traditional midwifes in the possible risks and in the detection of adverse events would be important.

#### 5.5.3. Pharmacovigilance Aspects

Due to the potential safety and toxicity issues that can be encountered with herbal products and the difficulty to establish quality specifications and controls [172], a specific pharmacovigilance system is recognized as important [173,174] and harmonizing national regulatory and programmatic pharmacovigilance efforts in Africa should be a priority. Pharmacovigilance should be integrated within communities and health facilities so that data concerning the composition, preparation, indications, and adverse effects of remedies can be collected [175]. The latency period between the use of an herbal product and the onset of an adverse event should also be determined, if possible, as this can make it easier to assess causality and propose eventual protective measures [172].

## 6. Deficiencies, Development Challenges and Possible Strategies

A number of important measures were put into place, and member states in the African area developed national policies and regulatory frameworks for traditional and complementary medicine practice, practitioners, and products between 2005 and 2018. As of 2018, the region outperformed the worldwide scenario in nearly every metric, with the exception of the regulation and registration of herbal medicines, where the proportion of member states in the region lagged behind that of all member states [176]. There are significant disparities in legislation and a wide range of procedures that must be followed when integrating medicinal plants into the traditional healthcare system. In reality, every nation has a kind of national authority; while phytomedicines are widely accepted in certain nations, they are regarded as foods in others, and their medicinal claims are not permitted [176]. There is also an array of different factors that might clarify these discrepancies among countries. The authorizations are specifically intended for the treatment and management of major and priority pathologies, including HIV/AIDS, respiratory conditions, hepatitis, malaria, and hypertension [177]. Among further variables, there is a lack of written scientific record, a lack of a national or regional pharmacopeia produced in this region of Africa, and a lack of a comprehensive monograph of the most significant medicinal plants [178]. One other possible explanation for the underutilization and paucity of research on therapeutic plants in several African nations could be their preference for exports; for example, Cameroon and Egypt claim to be the two largest African exporters of medicinal herbs [179]. Considering such unawareness of African women’s health practices, it is important to take measures to understand, concretize, facilitate (or sometimes discourage) their traditional uses of herbal medicines, in particular by encouraging researchers from all countries to conduct in-depth ethnobotanical studies on medicinal plants used by African women, both to compile information and to safeguard the traditional knowledge of the remedies applied to their specific health problems. It necessary to perform comparative studies between different regions and countries for the eventual converging of data that confirm the role of each plant and document their efficacy and safety. Recent years indicate major steps in this direction. In 2018, the research on traditional medicine attracted the interest of at least 34 research institutes in 26 African countries, compared to 18 and 20 in 2000 and 2012, respectively [178].

The challenges and possible approaches to merging traditional medical procedures with contemporary healthcare systems are numerous. They point to a need for developing coherent national policies in traditional medicine with efforts in healer recognition and accreditation, information, training, education, and communication. All parties involved in the health sector should receive training to develop platforms for cooperation and partnerships with reference laboratories and institutes which will help in mastering both practicians, practices, and products [176].

## 7. Conclusions

In an original approach, the present review attempted to compile the herbal treatments traditionally applied to all gender-specific health problems affecting African women, with emphasis on menstrual disorders, pregnancy-related problems, infertility, breast cancer, and postpartum care. A comprehensive approach allowed for a literature search, combining pertinent keywords to gather data from ethnobotanical surveys carried out throughout different parts of Africa. In most of the rural African population, traditional medicine and traditional midwifes are regarded as the primary healthcare choice for maternity [180] but some remedies are also recommended for less-common troubles. Indeed, traditional birth attendants and herbalists are freely accessible and there is a lot of confidence and respect among African women, with the perception that birth attendants are responsible for maintaining cultural traditions, which is considered a “must” for the baby. A widespread usage is recorded in many nations for almost 200 medicinal plants; some of the plants frequently used for women’s health include *Ricinus communis* (castor bean), *Cucurbita pepo* (pumpkin), *Zingiber officinale* (ginger), and *Allium sativum* (garlic). This review offers insightful information about African women’s traditional knowledge and practices, but also highlights important research gaps, such as the dearth of data from numerous African nations and the need for more thorough studies on the effectiveness and safety of these traditional remedies, and the hazards of toxicity and over- or under-dosage, particularly in pregnancy or otherwise vulnerable conditions. So far, the majority of reported investigations are only surveys or cross-sectional studies focusing on pregnancy. Lists of specific plants to avoid have been compiled as mother and newborn morbidity and mortality imply long-term detrimental health impacts. The identification of the responsible molecules and the estimation of the doses that would be safe are however still lacking. It is worth noting that the published works are often of low and median quality, medically speaking, and probably important methodological biases are rarely addressed. A rational use of the herbal medicines in African countries is also hampered by significant research gaps and a lack of a regulatory framework. There are also very few studies of women in the African diaspora who are likely to use such remedies, often in secret from their gynecologist.

## Figures and Tables

**Table 1:** Natural remedies used by African women during puberty.

County or Region	Used Remedy	Reference
Burkina Faso	An infusion of daaga (*Kalanchoe pinnata* (Lam.) Pers.) and kalakoe (*Ageratum conyzoides* Sieber ex Steud.).	[32]
Gabon and the Central African Republic	An infusion of papaya (*Carica papaya* L.), parsley (*Petroselinum crispum* (Mill.) Nyman ex A. W. Hill), and lemon (*Citrus limon* (L.) Osbeck).	[33]
Côte d’Ivoire and Benin	Caraway (*Carum carvi* L.), yarrow (*Achillea millefolium* L.), white willow (*Salix alba* L.), and peony (*Paeonia officinalis* L.).	[34]
Maghreb	Infusions of *Verbena officinalis* L., valerian (*Valeriana officinalis* L.), and rosemary (*Rosmarinus officinalis* L.) to relieve premenstrual syndromes.	[34]
Mali	Boiled foléré (*Hibiscus sabdariffa* L.), massep (*Ocimum gratissimum* L.), and parsley (*Petroselinum crispum* (Mill.) Nyman ex A. W. Hill) for irregular menstruation	[34]
Côte d’Ivoire	*Aloe vera* (L.) Burm.f. gel applied to the intimate area by women to treat infections.	[34]
Cameroon	Cinnamon infusion (*Cinnamomum verum* J. Presl) applied to the intimate area by women in case of infection.	[34]
Democtratic republic of the Congo	Papaya leaves (*Carica papaya* L.) and sombe (*Manihot esculenta* Crantz).	[35]
Burkina Faso, Guinea, Mali, and Nigeria	Infusion of Senegal rosewood (*Pterocarpus erinaceus* Poir.).	[36]
South Africa	Motherwort (*Leonurus cardiaca* L.) for irregular menstruation.	[37]
South Africa	Yarrow infusion (*Achillea millefolium* L.) for intimate hygiene problems.	[37]
Angola	Zimbabwe raspberry (*Rubus idaeus* L.) leaves for irregular menstruation.	[38]
Maghreb	Heavy periods are treated with a decoction of *Ligusticum wallichii* Franch. (a synonym of *Ligusticum striatum* DC.), peony (*Paeonia officinalis* L.), and nettle (*Urtica dioica* L.).	[39,40]
Burkina Faso, Côte d’Ivoire, Ghana, Guinea Bissau, Guinea, Nigeria, and Sierra Leone	The root of pea cane (*Phyllanthus niruri var genuinus* Mull Arg, synonym of *Phyllanthus amarus* Schumach. & Thonn.) is infused during heavy periods.	[41]
Sub-Saharan Africa, such as Gabon, Benin, and Côte d’Ivoire	The application of fintinko (*Harrisonia abyssinica* Oliv.) leaves with seeds, kaolin, and salt for intimate hygiene problems.	[42]
Sub-Saharan Africa, such as Gabon, Benin, and Côte d’Ivoire	Decoction and infusion of wal-biisem (*Euphorbia hirta* L.); yulin-gnuuga (*Ocimum gratissimum* L.); kultãnga (*Cassia alata* L., synonym of *Senna alata* (L.) Roxb.); goya (*Psidium guajava* L.); nekiljem (*Holarrhena floribunda* T. Durand and Schinz); rõbré (*Ageratum conyzoides* Sieber ex Steud.); and imbuur (*Citrus aurantifolia* (Christm.) Swingle) for intimate hygiene problems.	[43]
Senegal and Ghana	Parsley (*Petroselinum crispum* (Mill.) Nyman ex A. W. Hill) and garlic (*Allium sativum* L.) for intimate hygiene problems.	[44]

**Table 3:** Toxicological effects of some medicinal plants grown or cultivated in Africa.

Plant Name	Adverse Effects	References
Rosary pea (*Abrus precatorius* L.)	May induce abortion.	[153]
Chaff-flower (*Achyranthes aspera* L.)	Abortifacient activity in rats.	[154]
Aloe vera (*Aloe barbadensis* miller)	Latex may cause stimulation of uterus contraction and abortion.	[155]
Garlic (*Allium sativum* L.)	Excessive consumption may augment the threat of pregnancy loss, uterine contraction, and hemorrhage.	[156]
Betel-nut palm (*Areca catechu* (Linn))	Induced abortion.	[157]
Coffee senna (*Cassia occidentalis* L.)	High dose induced tissue damage in the mother and myocardium bone necrosis.	[158]
Chamomile (*Chamaemelum nobile* (L.) All.)	It is a stimulant of uterine contraction care.	[159]
Drumstick tree (*Moringa oleifera)*	Has abortifacient activity in albino rats.	[160]
Gopo berry (*Phytolacca dodecandra* L.)	Abortifacient activity.	[161]
Red paucipan (*Plumeria rubra* L.)	Reduced fetuses lives and induced labor.	[162]
*Ricinus communis* L. (Euphorbiaceae)	It can stimulate the passage of meconium and results in neonatal respiratory distress aspiration.	[163]
Rue (*Ruta graveolens* Linn.)	Stimulates uterus motility, abortion, and induces abnormal embryos.	[164]
Candle bush (*Senna alata* L.)	Abortifacient activity.	[165]
Fenugreek (*Trigonella foenum-graecum* L.)	Teratogenic effects, and seed consumption during pregnancy has been associated with a range of congenital malformations.	[158]
Valerian (*Valeriana officinalis* L.)	Its consumption at the second trimester of pregnancy can cause zinc deficiency in the fetus.	[166]
Chaste tree (*Vitus agnus-castus* L. (VAC)	Induces ovarian hyperstimulation and may enhance the threat of miscarriage.	[167]
